# *E. hellem* Ser/Thr protein phosphatase PP1 targets the DC MAPK pathway and impairs immune functions

**DOI:** 10.26508/lsa.202302375

**Published:** 2024-01-10

**Authors:** Jialing Bao, Yunlin Tang, Yebo Chen, Jiangyan Jin, Xue Wang, Guozhen An, Lu Cao, Huarui Zhang, Gong Cheng, Guoqing Pan, Zeyang Zhou

**Affiliations:** 1 The State Key Laboratory of Resource Insects, Southwest University , Chongqing, China; 2 Chongqing Key Laboratory of Microsporidia Infection and Control, Southwest University , Chongqing, China; 3 Tsinghua University-Peking University Joint Center for Life Sciences, School of Medicine, Tsinghua University, Beijing, China

## Abstract

Microsporidia are difficult to be completely eliminated once infected, and the persistence disrupts host cell functions. Here in this study, we aimed to elucidate the impairing effects and consequences of microsporidia on host DCs. *Enterocytozoon hellem*, one of the most commonly diagnosed zoonotic microsporidia species, was applied. In vivo models demonstrated that *E. hellem*-infected mice were more susceptible to further pathogenic challenges, and DCs were identified as the most affected groups of cells. In vitro assays revealed that *E. hellem* infection impaired DCs’ immune functions, reflected by down-regulated cytokine expressions, lower extent of maturation, phagocytosis ability, and antigen presentations. *E. hellem* infection also detained DCs’ potencies to prime and stimulate T cells; therefore, host immunities were disrupted. We found that *E. hellem* Ser/Thr protein phosphatase PP1 directly interacts with host p38α (MAPK14) to manipulate the p38α(MAPK14)/NFAT5 axis of the MAPK pathway. Our study is the first to elucidate the molecular mechanisms of the impairing effects of microsporidia on host DCs’ immune functions. The emergence of microsporidiosis may be of great threat to public health.

## Abstract (precis)

Intracellular pathogen *E. hellem* suppressed host DCs’ immune functions via pathogen-derived serine/threonine protein phosphatase PP1 directly targeting the host p38α/MAPK pathway.

## Introduction

Microsporidia are a huge group of intracellular pathogens that infect all animals, from invertebrates to vertebrates including human beings (1, 2). At least 15 species can infect humans, and the four most common ones are *Enterocytozoon bieneusi* (*E. bieneusi*), *Encephalitozoon hellem* (*E. hellem*), *Encephalitozoon cuniculi* (*E. cuniculi*), and *Encephalitozoon intestinalis* (*E. intestinalis*) (3, 4). Immunocompromised individuals were believed to be more vulnerable to microsporidia infections (5). However, accumulating evidence showed that immunocompetent individuals would also be infected, and the outcomes are often asymptomatic and therefore lead to latent infections (6, 7, 8). For instance, one study conducted in Cameroon revealed that 87% of teenagers and 68% of healthy asymptomatic individuals actually had subclinical microsporidia infections and were shedding spores (9). In addition, co-infections of microsporidia with other pathogens such as HIV, cryptosporidia, and *Mycobacterium tuberculosis* are underestimated, but actually quite common and usually have exacerbated outcomes compared with single pathogen infection alone (10, 11). These findings revealed the incidences of latent infection and persistence of microsporidia in immunocompetent individuals are much higher than we used to think. Although the issues about the latent infection of microsporidia in immunocompetent individuals had long been underestimated, it should now be paid more attention because the wide existence of microsporidia in nature and the wide host ranges of them may lead to emerging infections and serious public health problems (8, 12).

As the obligate intracellular pathogen, microsporidia interact actively with the host cells, and researchers are very interested in elucidating how microsporidia modulate host cell functions, especially the immune cell functions. By far, most studies were carried out using genetically knockout or immunodeficient mice to increase microsporidia infection and colonization rates, and usually focused on host adaptive immunity (13, 14). Yet, using immunocompetent animal models and elucidating the roles of hosts’ sentinel innate immunity during microsporidia–host interactions are much in demand (15, 16).

DCs, the professional APCs and the bridge of innate and adaptive immunities, are found participating in defending microsporidia infections (17, 18, 19). Moretto et al showed that in vitro infection of DCs by *E. cuniculi* would activate CD8^+^ T cells and help to release IFN-γ, which is known to be cytotoxic to microsporidia. Bernal et al found that DC differentiation and production of pro-inflammatory cytokine IL-6 were interfered with *E. intestinalis* infections (14, 15). Because the maturation and differentiation of DCs influence host’s overall antimicrobial capability, DCs might be the master regulator of host immune responses against microsporidia and other pathogen co-infections (20, 21).

The MAPK signaling pathway is essential in regulating many cellular processes including inflammation and stress responses. Along the line of signal transduction, there are several key proteinases that can be modulated, thus affecting the signaling outcomes. For example, the p38a (MAPK14) and MAP kinase kinases in the MAPK pathway have been reported to be regulated by several pathogens and medicines (22, 23). In addition, NFAT5 (nuclear factors of activated T cells), one of the transcription factors downstream of the MAPK pathway, has been found to be essential for expressions of pro-inflammatory genes such as *IL-6*, *IL-2*, and *H2Ab* (24, 25, 26, 27).

Ubiquitous serine/threonine protein phosphatase (PP1) is a single-domain catalytic protein that is exceptionally well conserved in all eukaryotes, from fungi to humans, in both sequence and function (28). In the human body, PP1 is responsible for about 30% of all dephosphorylation reactions (29, 30). Pathogenic microbes may express PP1 as a modulator of the host. For example, *M. tuberculosis* expresses PknG within the host macrophage to regulate host protein phosphorylation and interferes with autophagy flux, thus greatly affecting host cell functions (31, 32). Intracellular pathogen microsporidia possess a reduced genome to bear only a minimum of essential genes. However, we are very excited to find the existence of the *PP1* gene in the microsporidia genome, indicating its indispensable roles in pathogen growth and in pathogen–host interactions. It is therefore of great interest to exploit the regulation effects of microsporidia PP1 on DC functions.

Here in the current study, we use the murine model and cells cultured in vitro to thoroughly investigate the influence of microsporidia infection on DCs, and elucidate the regulation mechanisms of microsporidia on DCs’ immune responses. Our study will provide a molecular basis for developing novel prevention strategies for microsporidia infection and prevalence.

## Results

### Persistent infection of *E. hellem* increases host disease susceptibility and disturbs DC populations

**Figure S1.**
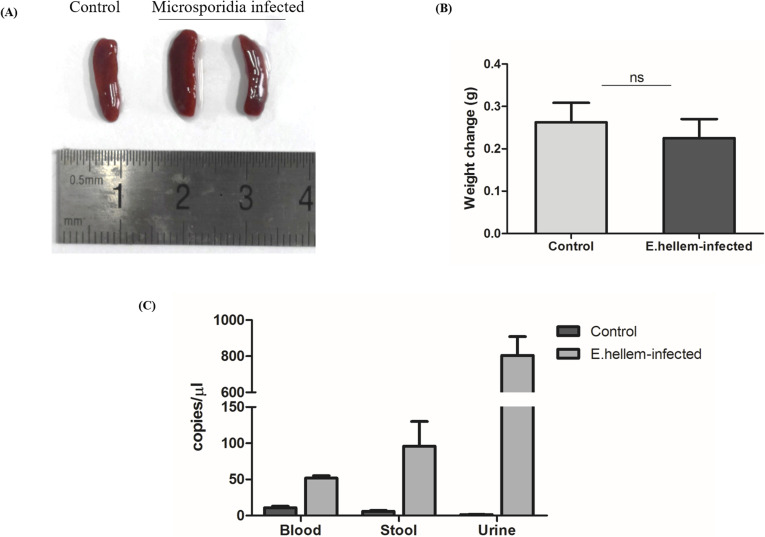
Proof of *E. hellem* persistence and unobvious manifestations. **(A)** Spleen size measurement showed no edema after microsporidia infections. **(B)** Body weights were measured and monitored until 17 dpi. Both uninfected and *E. hellem*-infected groups showed no body weight loss but slight body weight gain during this period (n = 10 per group; ns, no significance). **(C)** Persistence of *E. hellem* in the host. Peripheral blood, stool, and urine samples were taken on the 17 dpi from *E. hellem*-infected mice; uninfected mice were controls (n = 10 per group). The existence of *E. hellem* was verified by qRT-PCR using primers targeting the *E. hellem* SSU-rDNA region.

Microsporidia infections are usually asymptotic. However, the covert infection and persistence of microsporidia within hosts may disturb their immunity as a whole, making them more vulnerable to further challenges. Here in this study, we firstly used our previously established microsporidia infection model to infect WT C57BL/6 mice with *E. hellem*. As a result, *E. hellem* infections were covert as proved by no obvious symptoms such as spleen edema or significant weight loss (Fig S1A and B), but *E. hellem* could persist as proved by detection of the spores in host blood, stool, and urine samples even after more than half a month past infection (Fig S1C).

**Figure 1.**
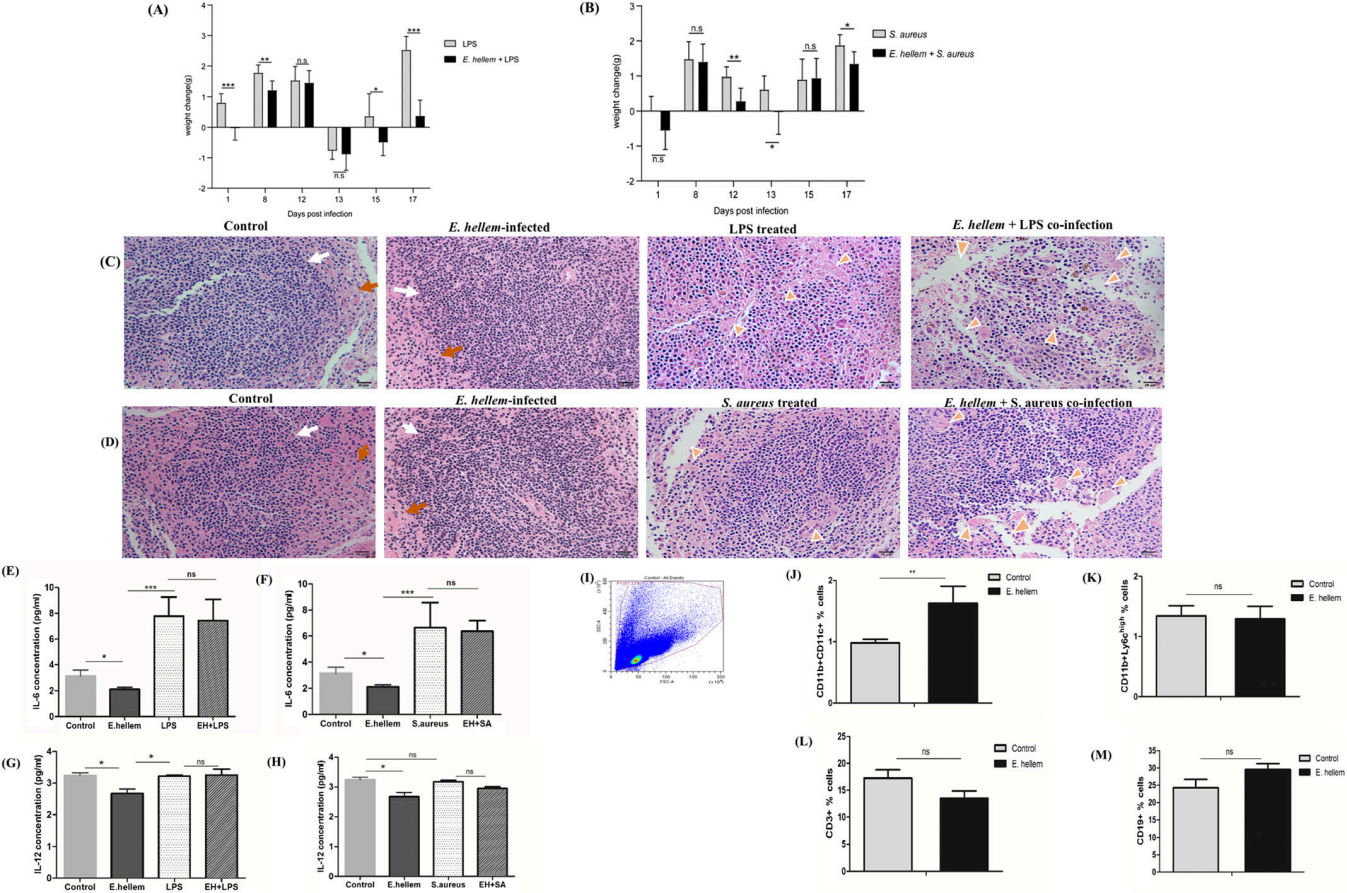
*E. hellem* infection and persistence increase host disease susceptibility and affect DCs. **(A)** LPS treatment in *E. hellem*–pre-infected mice (*E. hellem*+LPS) caused more body weight loss and slower/less body weight re-gain compared with LPS alone (n = 8 per group; * = *P* < 0.05, ** = *P* < 0.01, and *** = *P* < 0.001). **(B)** *S. aureus* infection in *E. hellem*–pre-infected mice (*E. hellem*+*S. aureus*) caused more body weight loss and slower/less body weight re-gain compared with *S. aureus* alone (n = 8 per group; * = *P* < 0.05, ** = *P* < 0.01, and *** = *P* < 0.001). **(C)** Hematoxylin–eosin staining of spleen samples after *E. hellem* infection alone had no obvious effects on tissue pathology compared with control, as white pulps (white arrows) and red pulps (orange arrows) arranged normally. However, *E. hellem* pre-infection plus LPS treatment caused more damages to tissues compared with LPS alone, as shown by more enlarged/distorted cells/cytosols and vacuolations (golden arrowheads) (scale bar = 20 μm). **(D)** *E. hellem* pre-infection plus *S. aureus* infection caused more damages to tissues compared with *S. aureus* alone, as shown by more enlarged/distorted cells/cytosols and vacuolations (golden arrowheads) (scale bar = 20 μm). **(E)** ELISA showed that the *E. hellem* infection (*E. hellem*) significantly down-regulated the expression of IL-6, compared with uninfected controls and LPS treatment (n = 8 per group; * = *P* < 0.05 and ** = *P* < 0.01). **(F)** ELISA showed that the *E. hellem* infection (*E. hellem*) significantly down-regulated the expression of IL-6, compared with uninfected controls and *S. aureus* infection (n = 8 per group; * = *P* < 0.05 and ** = *P* < 0.01). **(G)** ELISA showed that the *E. hellem* infection (*E. hellem*) significantly down-regulated the expression of IL-12, compared with uninfected controls and LPS treatment (n = 8 per group; * = *P* < 0.05 and ** = *P* < 0.01). **(H)** ELISA showed that the *E. hellem* infection (*E. hellem*) significantly down-regulated the expression of IL-12, compared with uninfected controls and *S. aureus* infection (n = 8 per group; * = *P* < 0.05 and ** = *P* < 0.01). All co-infection conditions, either *E. hellem* plus LPS (EH+LPS) or *E. hellem* plus *S. aureus* (*EH*+*SA*), did not arose higher but caused a slight lower level of cytokine levels compared with single challenges. **(I)** Flow cytometry analysis of the immune cell profiles from mouse mesenteric lymph nodes. Mesenteric lymph nodes were isolated from control or *E. hellem*-infected mice, and were teased into single-cell mixture (n = 3–5 lymph nodes from each mouse, eight mice per group). **(J)** Flow cytometry analysis showed (J) that DCs (CD11b+CD11c+) were significantly disturbed after *E. hellem* infection; **(K)** no significant changes in inflammatory monocytes (CD11b+Ly6c^high^); **(L)** no significant changes in T cells (CD3^+^); and **(M)** no significant changes in B cells (CD19^+^) (** = *P* < 0.01 and ns, no significance).

Interestingly, when the *E. hellem*–pre-infected mice were challenged with the secondary stimulus such as *Staphylococcus aureus* infection or LPS treatment, the hosts showed increased susceptibility to the secondary challenges. As shown, *E. hellem* pre-infection with secondary challenges together (mimicking co-infection conditions) would cause significantly more weight loss and slower weight re-gain in mice, compared with single challenges (Fig 1A and B). Similarly, *E. hellem* infection alone caused a limited extent of tissue damages in host organs, but the co-infection conditions lead to more irritations in tissues compared with single challenges alone (Fig 1C and D). In addition, ELISA analysis revealed that the worse outcomes of co-infection conditions would be due to the facts that *E. hellem* infection alone down-regulated the expressions of pro-inflammatory cytokines IL-6 and IL-12, and detained the surge of cytokine levels in the co-infection conditions (Fig 1E–H).

Because the major infection route of microsporidia is through ingestion, the assaulting pathogens were majorly sampled by phagocytes such as DCs in the intestinal mucosa and then drained to mesenteric lymph nodes (MLN). Therefore, we are very interested in investigating the modulation effects of *E. hellem* on the immune cell populations in the MLN. Flow cytometry analysis showed that DC populations were significantly altered after infection, but not the lymphatic T cells or B cells, nor the inflammatory monocytes (Fig 1I). Taken together, these data demonstrated that covert infection of *E. hellem* would persist within host, making host more vulnerable to further challenges (co-infection conditions), and the most affected sentinel cell populations are DCs; therefore, they may play key roles in microsporidia–host interactions.

### *E. hellem* interferes with DCs’ immune functions and maturation

**Figure 2.**
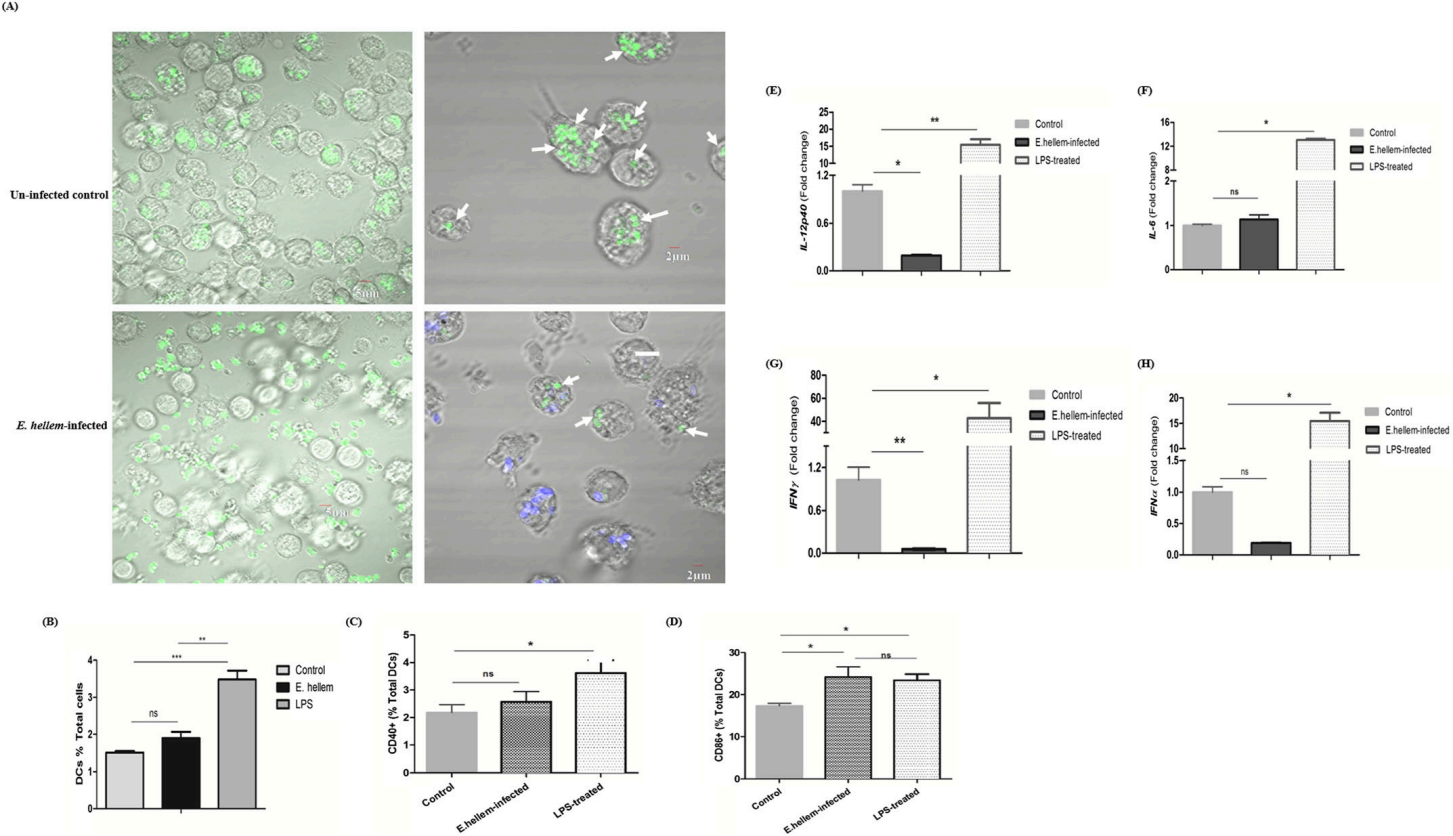
*E. hellem* interferes with DCs’ immune functions and maturation. **(A)** DCs were isolated from normal mice or *E. hellem*-infected mice, and fluorescent (GFP)-labeled *S. aureus* were then added to DC culture (MOI = 20:1). The phagocytic ability was assessed by fluorescent microscopy. Most of the added-in *S. aureus* (green) were engulfed by control DCs, whereas most added-in *S. aureus* (green) were floating outside of DCs isolated from *E. hellem*-infected mice (scale bar = 5 μm). Zoomed-in images showed that DCs from the *E. hellem*-infected group engulfed significant less *S. aureus* (green; arrows) compared with DCs from uninfected controls. Persistence of *E. hellem* spores was manifested by calcofluor white stain (blue) (scale bar = 2 μm). **(B)** Flow cytometry analysis showed that *E. hellem* infection retained the splenic DCs (33D1+) to the comparable level as the uninfected controls. Yet, LPS treatment significantly up-regulated the DCs (n = 8/group; ns, no significance, ** = *P* < 0.01, and *** = *P* < 0.001). **(C)** *E. hellem* infection, but not LPS treatment, inhibited the expressions of CD40 on DCs. The expression of CD86 was at a comparable level between *E. hellem* infection and LPS treatment, both significantly higher than uninfected control (n = 8/group; ns, no significance and * = *P* < 0.05). **(E)** Cytokine gene expression profiles from splenic DCs were assessed by qRT-PCR; results showed that the (E) *IL-12p40* expression level of DCs from *E. hellem*-infected mice was significantly down-regulated compared with uninfected controls, and was significantly lower than the LPS-treated ones. **(F)** *IL-6* expression level of DCs from *E. hellem*-infected mice was retained from uninfected controls, and was significantly lower than the LPS-treated ones. **(G)** *INF-γ* expression level of DCs from *E. hellem*-infected mice was significantly down-regulated compared with uninfected controls, and was significantly lower than the LPS-treated ones. **(H)** *IFN-α* expression level of DCs from *E. hellem*-infected mice was retained from uninfected controls, and was significantly lower than the LPS-treated ones (n = 8/group; ns, no significance, * = *P* < 0.05, ** = *P* < 0.01, and *** = *P* < 0.001).

The major immune functions of DCs include pathogen phagocytosis, cytokine expressions, and antigen presentation. Firstly, we isolated DCs from *E. hellem*-infected mice or controls, and compared their phagocytosis abilities with fluorescently labeled *S. aureus*. As shown, control DCs sufficiently engulfed fluorescent-labeled *S. aureus*, whereas the DCs from *E. hellem*-infected mice were reluctant to engulf *S. aureus* (Fig 2A and Video 1, Video 2, Video 3, and Video 4).

Video 1 Control DCs actively engulf *S. aureus*. Download video

Video 2 Closer look of engulfed *S. aureus* within DCs. Download video

Video 3 *E. hellem*–pre-treated DCs reluctantly engulf *S. aureus*. Download video

Video 4 Closer look of *E. hellem*-infected and packed DCs. Download video

Next, we assessed whether microsporidia infection affects the maturation capability of DCs. Spleens were collected from either *E. hellem*-infected, LPS-treated, or uninfected mice, respectively, processed as single-cell suspensions, and subjected to flow cytometry. Results showed that the matured DC populations were significantly increased after LPS treatment. Yet, the matured DC populations were at comparable levels between the *E. hellem*-infected group and uninfected controls (Fig 2B). Flow cytometry analysis also revealed that *E. hellem* detained the expressions of DC maturation and co-stimulatory surface markers, CD40 and CD86 (Fig 2C and D). Next, cytokine expressions of DCs were assessed by qRT-PCR analysis and we confirmed that DCs from the *E. hellem*-infected group expressed significantly lower levels of *IL-12p40*, *IFN-γ*, *IL-6*, and *IFN-α*, compared with DCs from uninfected controls or LPS-treated groups (Fig 2E–H).

Taken together, these findings demonstrated that *E. hellem* infection interferes with the full immune functions and full maturation processes of DCs, which would explain the above-shown detained cytokine levels in serum and increased host susceptibility to secondary pathogens.

### *E. hellem* detained DC antigen presentation and T-cell priming potencies

**Figure 3.**
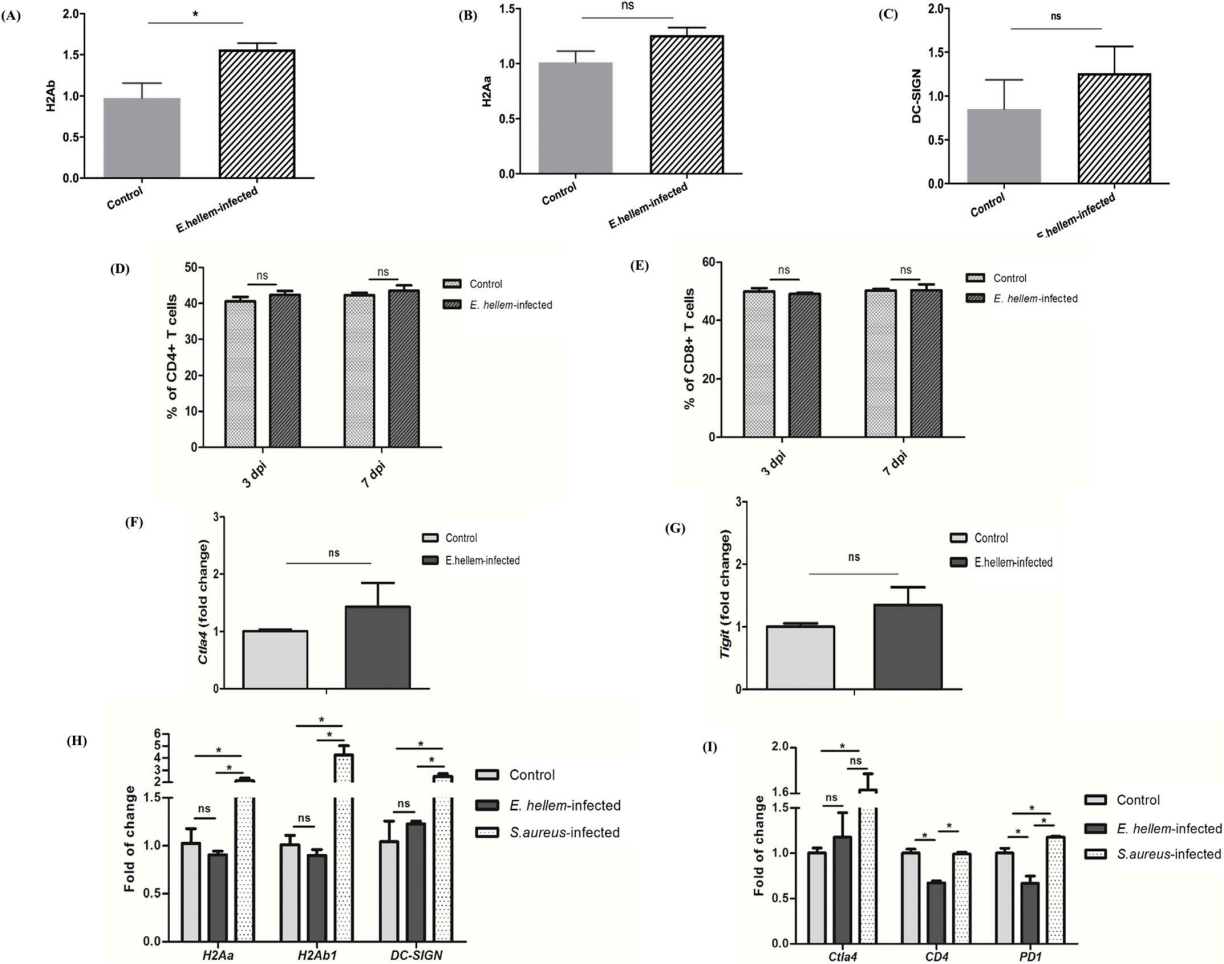
*E. hellem* detained the antigen presentation abilities and T-cell priming potencies of DCs. The expressions of DC antigen presentation–related surface markers were assessed by qRT-PCR assay. **(A)** Results showed that (A) *E. hellem* infection caused the up-regulation of H2Ab; **(B)** *E. hellem* infection detained the up-regulation of H2Aa; and **(C)** *E. hellem* infection detained the up-regulation of DC-SIGN (ns, no significance and * = *P* < 0.05). **(D, E)** Flow cytometry analysis of cell population changes after *E. hellem* infection showed that there were no significant stimulations or changes in populations of both (D) CD4^+^ T cells and (E) CD8^+^ T cells (n = 8/group; ns, no significance). **(F, G)** qRT-PCR analysis of the expressions of T-cell markers showed that neither (F) *Ctla4* nor (G) *Tigit* showed any changes after *E. hellem* infection (ns, no significance). **(H)** DC2.4 cells, either infected by *E. hellem* or *S. aureus*, or uninfected controls, were co-cultured with Jurkat T cells. The DC2.4 cells were isolated later for surface marker analysis. qRT-PCR results showed the representative antigen presentation markers were detained by *E. hellem* infection, compared with *S. aureus* infections. **(I)** Jurkat T cells isolated from co-culture with DC2.4 were also analyzed by qRT-PCR. Results confirmed that T cells co-cultured with *E. hellem*-infected DCs reluctant to up-regulate the *Ctla4*, and significantly down-regulated the expressions of T-cell activation surface markers *CD4* and *PD-1* (n = 10/group; ns, no significance and * = *P* < 0.05).

Fully functioning DCs would present the processed antigens to T cells and prime T-cell activation. Therefore, we analyzed the expressions of *H2Aa* and *H2Ab* in MHC-II, and *DC-SIGN* in the DCs isolated from either *E. hellem*-infected, LPS-treated, or uninfected mice, respectively. These molecules are essential for antigen presentation of DCs and T-cell priming (33). qRT-PCR assay showed that although *E. hellem* infection caused the up-regulation of *H2Ab*, it detained the up-regulation of both *DC-SIGN* and *H2Aa* in MHC-II (Fig 3A–C). These findings indicated that the antigen presentation abilities of DCs would be severely impaired by *E. hellem*. Next, the T-cell population alterations and stimulations were assessed by flow cytometry. Results showed that the neither CD4^+^ T cells nor CD8^+^ T cells showed significant stimulation after *E. hellem* infection (Fig 3D and E). qRT-PCR analysis of the expressions of *Ctla4* and *Tigit*, the known markers for T-cell activation, demonstrated that there was no significant activation of T cells after *E. hellem* infection (Fig 3F and G).

Next, we took one more step forward by co-culturing DC2.4 cells with Jurkat T cells in vitro, to further demonstrate the interference of *E. hellem* with DCs’ functions and reluctance of T-cell priming. Here, DC2.4 cells were infected by *E. hellem* or *S. aureus* and then co-cultured with Jurkat T cells in vitro for 12 h. DCs and T cells were then separated, and total RNAs were extracted for qRT-PCR analysis. Results again confirmed that the *E. hellem*-infected DCs were reluctant to up-regulate expressions of *H2Aa*, *H2Ab*, and *DC-SIGN* compared with *S. aureus*-infected controls (Fig 3H), whereas the T cells co-cultured with *E. hellem*-infected DCs detained or even significantly down-regulated the expressions of T-cell activation markers such as *CD4*, *Ctla4*, and *PD-1* (Fig 3I).

Taken together, our findings demonstrated that *E. hellem* infection defers DCs’ immune functions and T-cell priming potencies. The impairing effects of *E. hellem* on DCs explained previous phenomena that *E. hellem* pre-infection disrupted host immune responses and increased host vulnerability against further co-infections or challenges.

### The MAPK/NFAT5 signaling pathway is key for *E. hellem*–DC interactions and modulations

**Figure 4.**
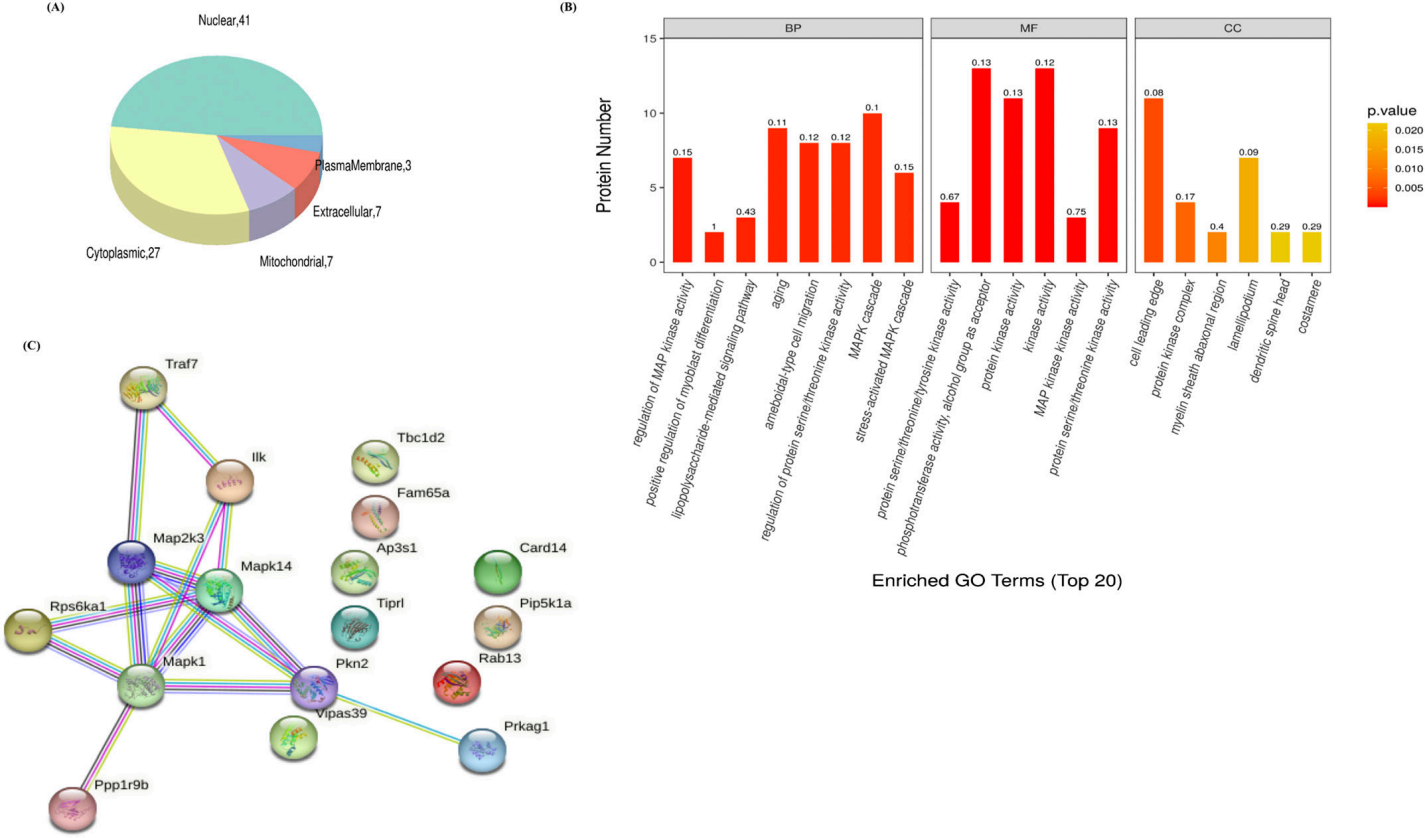
p38α/MAPK signaling pathway is key for *E. hellem*–DC interactions and modulations. **(A)** Pie chat of subcellular localization of top differentially expressed DC proteins after *E. hellem* infection. The top localization is cytoplasm and nucleus. **(B)** GO enrichment analysis of top differentially expressed DC proteins after *E. hellem* infection. Many signaling pathways including the MAPK pathway were among the most affected cellular events. **(C)** Protein–protein interaction network showed that top differentially expressed DC proteins were associated with the MAPK signaling pathway.

We have demonstrated that *E. hellem* infection caused down-regulation of several immune-functioning genes such as *IL-6*, *H2Ab*, and *H2Aa* in DCs. It is known that these genes’ expressions would all be regulated by the MAPK pathway, especially through the transcription factor NFAT5. We used mass spectrometry to evaluate the proteomic profile changes in DCs after *E. hellem* infection comprehensively; however, special attentions were paid to the intracellular signaling pathways in order to get better understandings of modulation mechanisms. Subcellular localization analysis revealed that most of the differentially expressed DC proteins were localized in the cytoplasm or nucleus (Fig 4A), confirming that pathogen–host interactions and modulations were indeed occurred mostly in the cytoplasm and consequently in the nucleus at transcriptional levels. Gene Ontology (GO) enrichment and Kyoto Encyclopedia of Genes and Genomes (KEGG) analyses further demonstrated that many cellular events including the MAPK signaling pathways/cascades were indeed affected by *E. hellem* infection (Fig 4B). To concretize, we listed the representative top differentially expressed DC proteins, which are associated with cellular responses, signal transduction, and transportation, in Table 1. (All significant differentiated DC proteins are listed in Table S1.) The protein–protein interaction network analysis of these representative top identified proteins revealed that the MAPK signaling pathway would be one of the central links of these dysregulated host proteins (Fig 4C), further confirming the key role of the MAPK/NFAT5 axis during pathogen–host interactions.

**Table 1.** Representative DC proteins differentially expressed after *E. hellem* infection.

Protein ID	Protein name	Gene name	Function	Alterations
P47811.3	Mitogen-activated protein kinase 14	Mapk14	MAP kinase p38, which acts as an essential component of the MAPK signal transduction pathway	Down
P63085	Mitogen-activated protein kinase 1	Mapk1	Serine/threonine kinase, which acts as an essential component of the MAP kinase signal transduction pathway	Down
O09110	Dual specificity mitogen-activated protein kinase kinase 3	Map2k3	Essential components of MAP kinase signal pathway, catalyzes the concomitant phosphorylation of a threonine and a tyrosine residue in the MAP kinase p38	Down
P18653	Ribosomal protein S6 kinase α-1	Rps6ka1	Serine/threonine protein kinase that acts downstream of ERK (MAPK1/ERK2 and MAPK3/ERK1) signaling and mediates activation of the transcription factors CREB1, ETV1/ER81, NR4A1, and so on.	Down
Q922B6	E3 ubiquitin-protein ligase TRAF7	Traf7	Auto-ubiquitination regulated by MAP3K3, potentiates MEKK3-mediated activation of the NF-kappa-B in signaling	Down
O55222	Integrin-linked protein kinase	Ilk	Act as a mediator of inside-out integrin signaling	Down
B1AVH7	TBC1 domain family member 2A	Tbc1d2	GTPase-activating protein for RAB7A and signaling effector	Down
Q9DCR2	AP-3 complex subunit sigma-1	Ap3s1	Facilitates the budding of vesicles from the Golgi membrane and may be directly involved in trafficking to lysosomes	Down
Q8BH58	TIP41-like protein	Tiprl	Allosteric regulator of serine/threonine protein phosphatase 2A (PP2A)	Down
Q8BGQ1	Spermatogenesis-defective protein 39 homolog	Vipas39	Involved in endosomal maturation and lysosomal trafficking	Down
Q6R891	Neurabin-2	Ppp1r9b	Scaffold protein in multiple signaling pathways.	Up
Q99KF0	Caspase recruitment domain–containing protein 14	Card14	Scaffolding protein that can activate the inflammatory transcription factor NF-kappa-B and p38/JNK MAP kinase signaling pathways.	Up
Q68FE6	Rho family–interacting cell polarization regulator 1	Ripor1	Effector protein for Rho-type small GTPases that plays a role in cell polarity, signaling, and directional migration	Up
O54950	5-AMP–activated protein kinase subunit γ-1	Prkag1	AMP/ATP-binding subunit of AMP-activated protein kinase (AMPK), a kinase that plays key roles	Up
Q8BWW9	Serine/threonine protein kinase N2	Pkn2	PKC-related serine/threonine protein kinase and Rho/Rac effector protein that participates in specific signal transduction during cellular signaling	Up
P70182	Phosphatidylinositol-4-phosphate 5-kinase type-1 alpha	Pip5k1a	Catalyzes the phosphorylation of PtdIns4P to form PtdIns (4, 5)P2, involves in cellular phagocytosis, migration, signaling.	Up
Q9DD03.1	Ras-related protein Rab-13	Rab13	Small GTPase key regulators of intracellular membrane trafficking	Up

Table S1 Top differentially expressed DC proteins after *E. hellem* infection.

**Figure 5.**
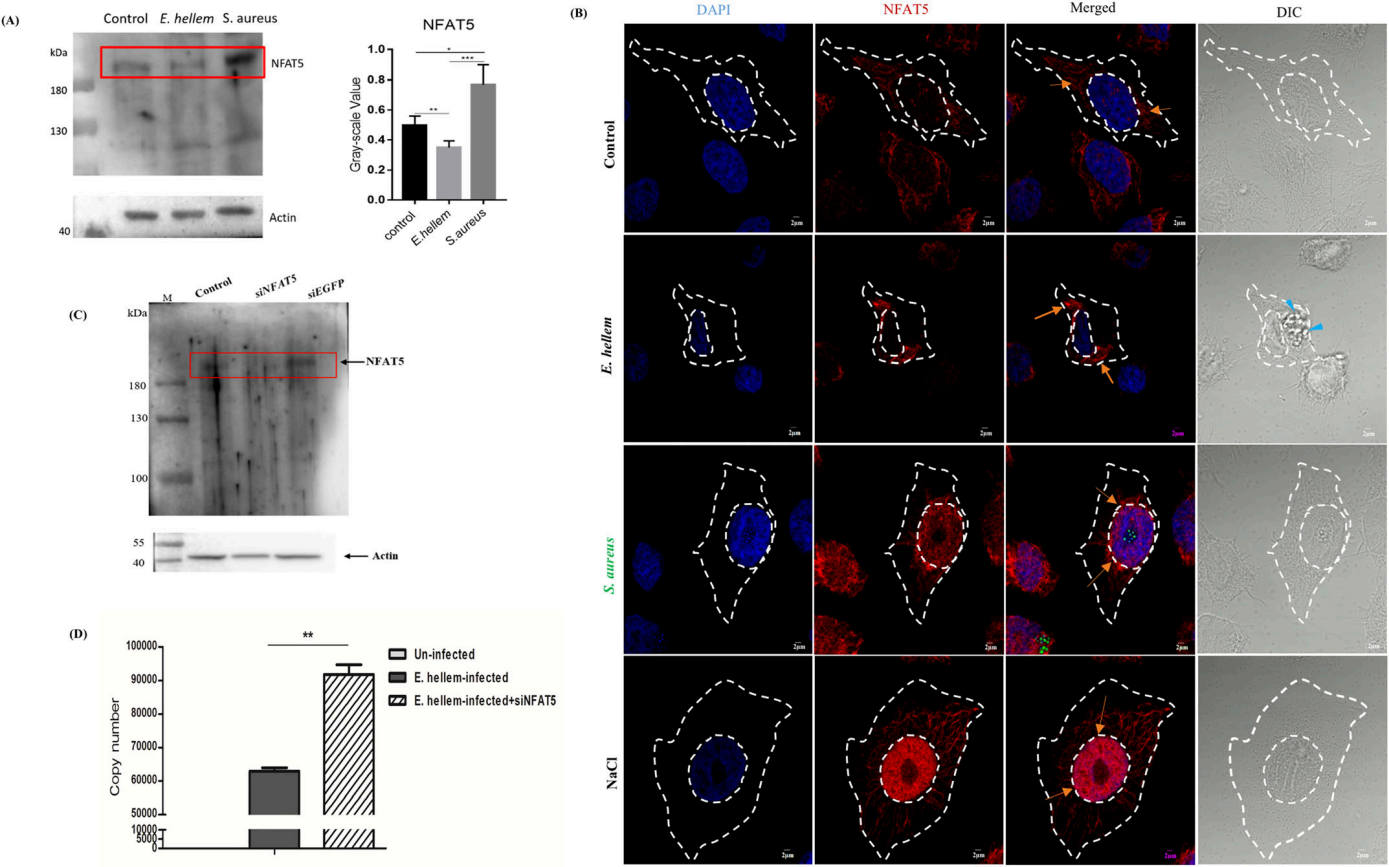
NFAT5/MAPK signal axis is essential during *E. hellem* infection and cellular modulation. **(A)** Western blot analysis of NFAT5 protein levels in DCs showed that NFAT5 expression was suppressed by *E. hellem* infection, compared with uninfected or *S. aureus* infection controls. **(B)** Immunofluorescent microscopy of NFAT5 localization in DCs. NFAT5 (Alexa Fluor 594) is constitutively expressed in the cytoplasm (orange arrows) of uninfected control DCs. The localization of NFAT5 was retained in the cytoplasm (orange arrows) after *E. hellem* infection. The *E. hellem* was able to proliferate in DCs and form parasitophorous vacuole (blue arrows). *S. aureus* infection and osmotic stimulation control (NaCl) were applied to DCs, and both can lead to up-regulation of NFAT5 expressions and re-localization into nucleus (orange arrows) (scale bar = 2 μm). **(C)** Western blot analysis showed that NFAT5 protein levels are significantly down-regulated after RNAi. **(D)** qRT-PCR analysis of *E. hellem* proliferation within host DC2.4 cells, as reflected by the copy numbers of *E. hellem*-specific *PTP4* (n = 8/group; ** = *P* < 0.01).

Next, we assessed the expressions and subcellular localization of the transcription factor, NFAT5, after *E. hellem* infection. Western blot assays showed that the NFAT5 expressions in DCs, from in vitro cell culture or from in vivo murine model, were suppressed by *E. hellem* infection (Fig 5A). An immunofluorescent assay was used not only to prove with visual evidence *E. hellem*’s persistence and proliferation within DCs, but also to demonstrate the inhibition effects of *E. hellem* on the translocalization of NFAT5 from cytoplasm into the nucleus (Fig 5B). To fully verify the essential roles of NFAT5 in DCs when combating *E. hellem*, we next knocked down the NFAT5 in DC2.4 cells by RNAi assay and demonstrated that the proliferation of *E. hellem* within DCs was increased (Fig 5C and D). These findings suggest that the NFAT5 and related MAPK signaling pathway is the key axis in responding to *E. hellem* infection, and therefore may be the major regulation targets of pathogen–host interactions.

### *E. hellem* serine/threonine protein phosphatase (PP1) targets DC p38α (MAPK14)/MAPK

To identify the effector proteins of *E. hellem* in regulating DC functions, we next examined the pathogen-derived proteins identified in *E. hellem*-infected DCs by mass spectrometry. All *E. hellem* proteins identified in DCs are listed in Table S2, and representatively top expressed *E. hellem* proteins are listed in Table 2. Analysis results revealed that most of these pathogen-derived proteins are associated with protein binding, cellular responses, and signal transduction, with the serine/threonine protein phosphatase PP1 as one of the top expressed *E. hellem*-derived proteins in DCs.

Table S2 *E. hellem*-derived proteins identified in infected DCs.

**Table 2.** Representative *E. hellem* proteins identified in infected DCs.

Protein ID	Gene ID	Protein name/function
XP_003888309.1	13466767	PP1 serine/threonine phosphatase
XP_003886772.1	13467540	Ras-like GTP-binding protein
XP_003887182.1	13468215	GTP-binding nuclear protein
XP_003887892.1	13466917	Rab GTPase
XP_003886950.1	13467595	Beta-tubulin
XP_003886661.1	13466496	Eukaryotic translation initiation factor 2 subunit gamma
XP_003886837.1	13467379	RAD3-like DNA-binding helicase
XP_003887105.1	13467910	Nop56p-like protein
XP_003887061.1	13468065	DNA topoisomerase II
XP_003886739.1	13467235	Dihydrofolate reductase
XP_003887411.1	13467332	Histidyl-tRNA synthetase
XP_003887230.1	13468325	Hypothetical protein
XP_003887352.1	13467170	Hypothetical protein
XP_003887011.1	13467738	Hypothetical protein
XP_003887459.1	13467461	Hypothetical protein

**Figure 6.**
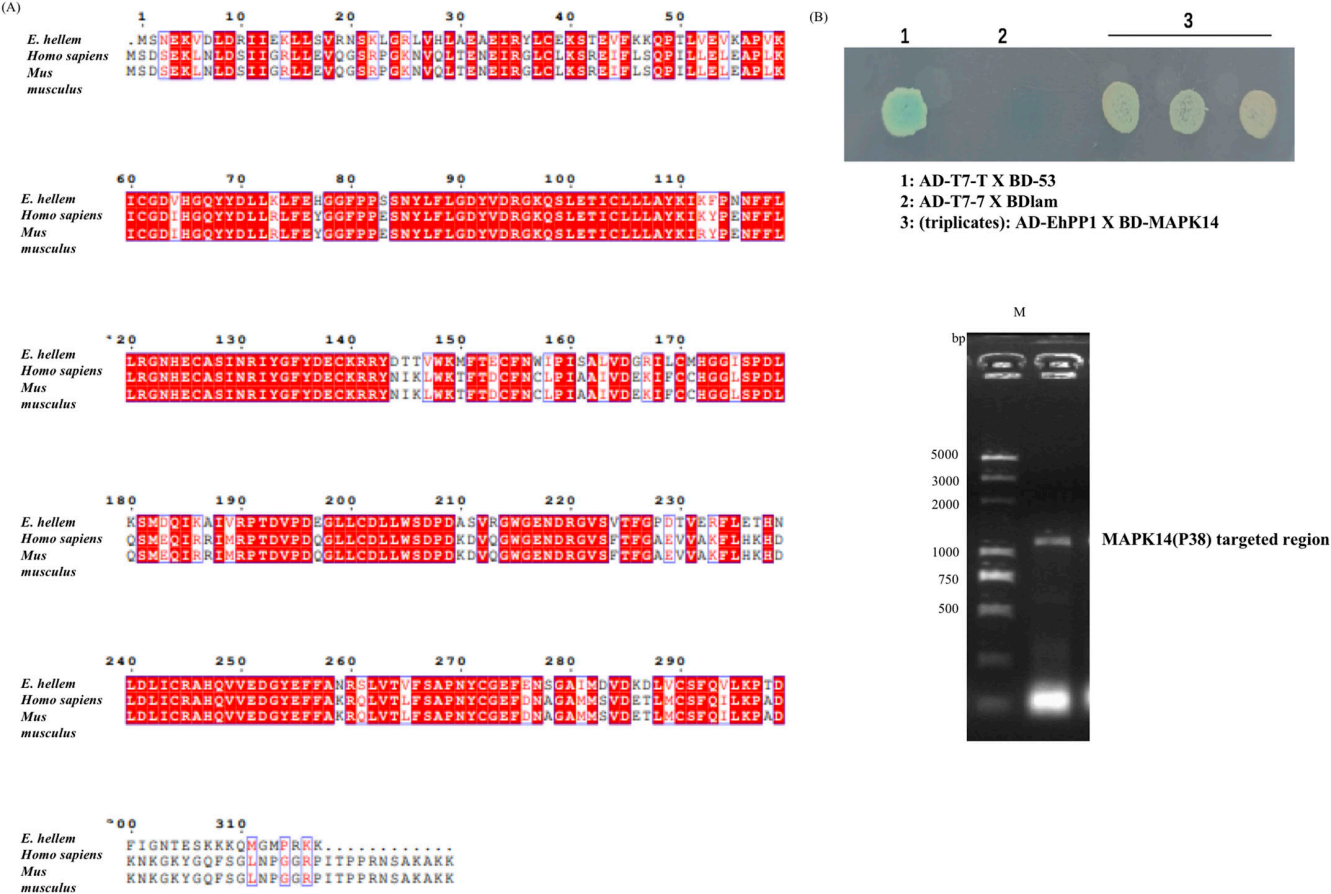
Direct binding of *E. hellem* PP1 with DC p38α(MAPK14). **(A)** Protein sequence alignments of serine/threonine protein phosphatase (PP1), derived from *E. hellem* (XP_003888309.1), human (P62136.1), and mouse (P62137.1). Alignments confirmed that PP1s, especially at the catalytic regions, were highly conserved among different species. **(B)** Yeast two-hybrid assay. DC MAPK14 was cloned into pGBKT7 plasmid (BD-MAPK14), and *E. hellem*–serine/threonine protein phosphatase PP1 was cloned into pGADT7 plasmid (AD-EhPP1). The plasmids were transformed into competent yeast cells, and the binding was validated in synthetic dropout-Leu-Trp-Ade-His medium supplemented with Xα-gal. The fusion strain of pGBKT7-53 with pGADT7-T was used as a positive control, and the fusion strain of pGBKT7-lam with pGADT7-T was used as a negative control. The EhPP1 and MAPK14 fused clones were subjected to PCR and gel electrophoresis to confirm the existence of target sequences.

**Figure 7.**
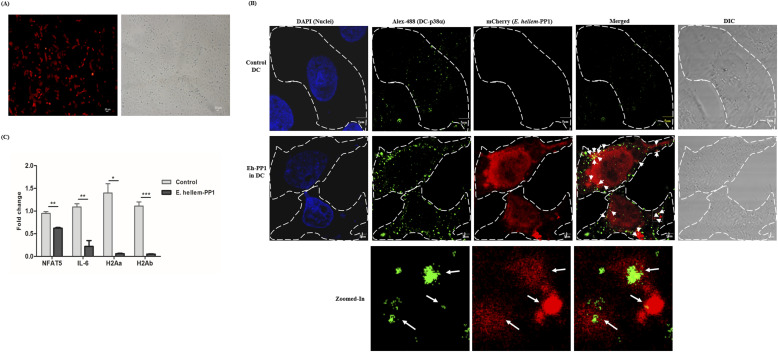
Direct interaction of *E. hellem* PP1 with DC p38α (MAPK14) and the down-regulated MAPK pathway gene expressions. **(A)** Expressions of *E. hellem* PP1 in DCs. Fluorescent microscopy confirmed the expression of *E. hellem*-derived PP1 (pCMV-mCherry-PP1) in the cytoplasm of DCs (red color) (scale bar = 20 μm). **(B)** Immunofluorescent microscopy confirmed the co-localization of *E. hellem* PP1 with DC p38α (MAPK14). Heterologously expressed *E. hellem*–PP1 (mCherry, red color) is expressed in the cytoplasm of DCs. The DC p38a (MAPK14) is labeled as Alexa Fluor 488 (green color) and is constitutively expressed in the cytoplasm of DCs. *E. hellem* PP1 co-localized with DC p38α (MAPK14) (arrows) (scale bar = 5 μm). **(C)** qRT-PCR analysis of DC *NFAT5*, *IL-6*, *H2Aa*, and *H2Ab* of MHC-II. All these genes were significantly down-regulated when DCs have heterologously expressed *E. hellem* PP1 (n = 12/group; * = *P* < 0.05, ** = *P* < 0.01, and *** = *P* < 0.001).

PP1 is a major Ser/Thr phosphatase and highly conserved in all eukaryotes, and is known to regulate MAPK signaling at several levels including direct interaction with components such as p38α in the pathway. To investigate whether *E. hellem*-derived PP1 could directly interact with DC p38α, we used yeast two-hybrid assay and confirmed that *E. hellem* PP1 directly interacts with DC P38α (MAPK14) (Fig 6). Moreover, we expressed *E. hellem*-derived PP1 in normal DCs (Fig 7A) and proved that the heterologously expressed *E. hellem* PP1 co-localized with host p38α (MAPK14) (Fig 7B). More importantly, the expressions of immune-functioning molecules such as *NFAT5*, *IL-6*, *H2Aa*, and *H2Ab* were significantly down-regulated by expressing *E. hellem* PP1 in DCs (Fig 7C). These findings demonstrated that the inhibiting effects of *E. hellem* on DCs’ immune functions were mostly exerted by its serine/threonine protein phosphatase PP1.

**Figure 8.**
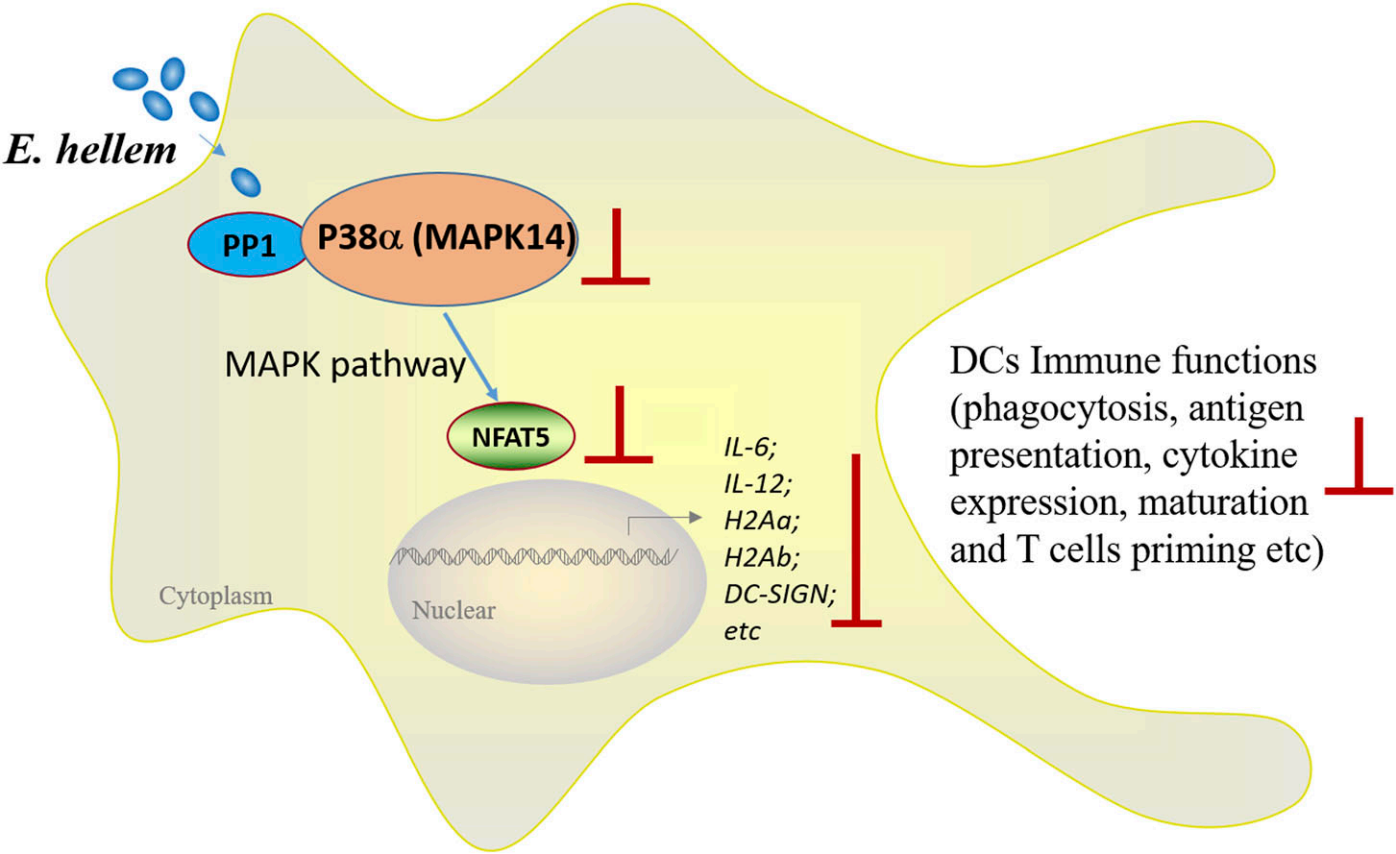
Image illustration and summary of *E. hellem* expressed the serine/threonine protein phosphatase PP1, directly interacted with DC MAPK14. The direct interaction would interfere with normal functions of MAPK14 and the subsequent transduction of the MAPK pathway; therefore, the downstream transcription factor NFAT5 expression and localization were disrupted. Eventually, various immune-related genes of DCs were down-regulated and the immune functions of DCs were severely impaired.

All taken together, this is the first clear evidence that microsporidia-derived proteinase directly targets and modulates the MAPK signaling pathway, thereby impairing DCs’ immune functions. Consequently, both innate and adaptive immune responses are suppressed and the host becomes more vulnerable against further pathogen infections and co-infections (Fig 8).

## Discussions

Our study is the first to elucidate the molecular mechanism of microsporidia manipulation of host DCs, and more importantly to prove the damaging effects of microsporidia persistence on host immune systems and the increased chances of being co-infected by other pathogens. In this study, we used the commonly diagnosed and zoonotic species of microsporidia, *E. hellem*, as the pathogen. In the MLN of *E. hellem*-infected C57BL/6 mice, we found that DCs, but not monocytes nor lymphocytes, were the most disturbed immune cell group. In vitro and in vivo analyses confirmed that the DCs’ immune functions were severely suppressed and impaired after *E. hellem* infection, reflected by the phagocytosis ability, maturation process, cytokine expressions, and T-cell priming potentials. To identify the regulation mechanisms, mass spectrometry and yeast two-hybrid analyses were applied. These analyses demonstrated that the DC MAPK/NFAT5 axis was essential for pathogen–host interaction and *E. hellem* serine/threonine protein phosphatase PP1 directly interacts with p38α (MAPK14) to manipulate DCs’ immune functions. RNAi assays showed that knocking down of NFAT5 in the MAPK/NFAT5 axis leads to increased proliferation of *E. hellem* within host, confirming the key function of MAPK signaling pathways during pathogen–host interactions and providing possible targets of disease prevention and control.

The MAPK pathways are ubiquitous and highly conserved in all eukaryotes and important for the regulation of various biological processes (34, 35). In immune cells, the MAPK pathway is associated with regulating the production of inflammatory and anti-inflammatory cytokines, cell viability, and the function of APCs (36). Interestingly, the MAPK pathway was found to be totally lost in microsporidia species (37). In our study, we demonstrated by mass spectrometry that *E. hellem* infection disturbed the proteins in the host’s MAPK pathway, such as p38α(MAPK14), MAPK1, and Map2k3. Therefore, the pathogen could target one or many of them to manipulate the host signal transduction pathway without affecting itself. In fact, we proved the direct interaction between pathogen-derived serine/threonine protein phosphatase 1 (PP1) and p38α(MAPK14). The direct interaction indeed impaired the whole transduction process and the downstream outcomes, as we demonstrated in this study that the expressions of transcription factors and pro-inflammatory genes/cytokines of DCs were severely detained or inhibited. Besides the proteins in the MAPK pathway, we also identified other differentially expressed proteins such as neurabin-2, ribosomal protein S6 kinase α-1, and E3 ubiquitin-protein ligase TRAF7. These proteins are functioning as scaffold proteins or in other cellular pathways such as ERK and ubiquitination (38, 39, 40). It is thus reasonable to infer that these different signaling pathways were cross-linked together in responding to *E. hellem* infection and modulation. In addition to cross-talks, we are also very interested in identifying which pattern recognition receptors were responsible for reception of *E. hellem* and activation of MAPK signaling in the upstream. Actually, the mass spectrometry analysis provided some candidates such as the integrin-linked protein kinase, which is an adaptor of integrin-related signal reception and transduction (41).

The essential roles and the manipulations of DCs during *E. hellem*–host interactions have been elucidated in our study. Yet, we should not neglect the involvement of other immune cells and processes. For instance, the involvement and protective roles of CD8^+^ T cells against microsporidia infection have been demonstrated in a mouse model study (42). These findings were in accordance with our findings that when the T-cell priming capabilities were disrupted in DCs, less of effector T cells such as CD8+T cells would be stimulated; therefore, the host had worse outcomes from *E. hellem* infection and increased susceptibility to co-infecting secondary pathogens. In addition, the infection of microsporidia in other immune cells may also manipulate normal cell functions. For instance, it is known that p38α (MAPK14) regulates the oncogenic process, autophagy, and apoptosis of many cells such as inflammatory monocytes and B lymphocytes (43, 44). The microsporidia persistence within those cells may not affect immune responses as they do in DCs, but may arose other problems such as increased autophagy or apoptosis so that the whole immune systems were compromised. Therefore, it is of great importance to fully investigate the effects of microsporidia on host cells in order to prevent microsporidiosis and consequent co-infections.

Serine/threonine PP1 is a highly conserved protein phosphatase in all eukaryotes, which regulates critical cellular processes including cell cycle progression, apoptosis, and metabolism. In mammalian cells, the involvement of PP1 in several oncogenic pathways has become evident and has been recognized as a potential drug target in cancer (45). In eukaryotic pathogens, the important regulation roles of PP1 are getting more attentions in recent years. For instance, PP1 has been found to regulate pathogen cell maturation, proliferation, and metabolism (46, 47). As a result, the existence of pathogen-derived PP1 within host cells may be of great importance for both pathogen cells’ intracellular proliferation and host cell manipulation. As for microsporidia, many species have been proved to possess this proteinase and exert important functions, for example, in germination (48, 49). Our study is the first and very excited one to identify the *E. hellem*-derived serine/threonine PP1 and verify the direct interaction with host p38α (MAPK14) in the MAPK signaling pathway. Interestingly, it is reported that PP1 would also interact and dephosphorylate RPS6KB1, homologous to our identified ribosomal protein S6 kinase α-1. Considering the intrinsic cross-talks and links of these host proteins, it will be not surprising to identify multiple effects of microsporidia-derived PP1 on host immune responses and other cellular processes in future studies.

Microsporidia were used to be considered as opportunistic pathogens, exist widely in nature, and have long been underestimated by their limited symptoms. However, we proved in this study that the persistence of microsporidia within host and co-infections with multiple pathogens would become a great threat to public health, as they impair host’s innate and adaptive immunities and increase the host susceptibility to other pathogens. Therefore, microsporidia detection, prevention, and control should get enough attentions in the future.

## Materials and Methods

### Pathogens

The *Encephalitozoon hellem* (*E. hellem*) strain (ATCC 50504) is a gift of Prof. Louis Weiss (Albert Einstein College of Medicine, USA). Spores were inoculated and propagated in rabbit kidney cells (RK13; ATCC CCL-37), and cultured in Eagle’s MEM with 10% FBS (Thermo Fisher Scientific). The spores were collected from culture media, purified by passing them through 5-μm-size filter (Millipore), and stored in sterile distilled water at 4°C (50). Spores were counted with a hemocytometer before usage.

*S. aureus* (*S. aureus*) was gifted by Dr. Xiancai Rao (Department of Microbiology, College of Basic Medical Sciences, Army Medical University, Chongqing, China). The microbe was modified on the basis of strain of N315 to express EGFP for visibility during observations, and cultured on TSB medium (51).

### Animals

WT C57BL/6 mice (6 wk, female) were reared in an animal care facility according to the Southwest University–approved animal protocol (SYXK-2017-0019). At the end of the experiment, all mice were euthanized using carbon dioxide narcosis and secondary cervical dislocation.

### Cells and cell lines

The primary DCs were isolated from mouse MLN, spleen, and bone marrow. Mesenteric lymph nodes aligned with the intestine were moved by forceps, washed with cold PBS, and teased into single-cell mix in harvesting medium such as RPMI 1640. Bone marrow cells were flushed by RPMI 1640 from the femur of dissected mice. Mouse spleens were digested in spleen dissociation medium (STEMCELL Technologies) at room temperature, followed by gently passing several times through a 16 Gauge blunt-end needle attached to a 3-cc syringe and then through a primed 70-μm nylon mesh filter. The single-cell suspension, from either bone marrow or spleen, was then subjected to EasySep Mouse Pan-DC Enrichment Kit (STEMCELL Technologies) to isolate DCs only. Briefly, Enrichment Cocktail and subsequently Biotin Selection Cocktail were added to the sample. Next, the sample was incubated with magnetic particles, and a magnet was used to negatively select the DC portion. The isolated DCs were counted and cultured in RPMI 1640 (supplemented with 10% FBS and penicillin/streptomycin) (Gibco) in a 37°C, 5% CO_2_ incubator.

A DC line, DC2.4 (SCC142; Sigma-Aldrich), was purchased from BeNa Culture Collection, China. The cells were cultured in RPMI 1640 supplemented with 10% FBS and penicillin/streptomycin (Gibco) in a 37°C, 5% CO_2_ incubator.

The suspension T-cell line, Jurkat cell (clone E6-1; ATCC), was purchased from Fudan University IBS Cell Center, China. The cells were cultured in RPMI 1640, supplemented with 10% (vol/vol) FBS and 1% (vol/vol) penicillin/streptomycin (Gibco) in a 37°C, 5% CO_2_ incubator.

### Microsporidia infection

In vivo infection of microsporidia to WT mice was achieved as follows. 1 × 10^7^ *E. hellem* spores/mouse/day were inoculated into WT mice for 2 d, and the mice were transiently pre-treated with dexamethasone (Cas 2392-39-4; Aladdin) to increase infection rates, but no significant effects on immune cells were assessed in our established murine model and other reports (52, 53, 54, 55). The control groups were treated with either PBS, LPS (5 mg/kg), or *S. aureus* (1 × 10^7^ CFU/mouse). The body weights were monitored. At the endpoint of the experiment, mice were euthanized by CO_2_ inhalation. Samples of blood, urine, feces, and organs were collected for further investigations.

In vitro infection of microsporidia was achieved by adding *E. hellem* spores (30:1/spores: cells) to primary DCs or DC2.4 cell cultures.

Successful invasion and colonization of *E. hellem* within cells or in host organs could be verified by immunofluorescent assays or qRT-PCR using *E. hellem* primers targeting the conserved SSU-rDNA (5′-TGAGAAGTAAGATGTTTAGCA-3′; 5′-GTAAAAAGACTCTCACACTCA-3′) (5).

### *S*. *aureus* infection

For in vivo infection, the bacteria were inoculated into mice via intraperitoneal injection at the dose of 10^7^ CFU/mouse. For in vitro infection, the bacteria were added to cell cultures at the ratio of 5:1 (DCs: *S. aureus*) or at the ratio of 1:1 (DCs: T cells), for various experimental purposes.

### Co-culture of DCs with T cells

DCs were infected with *E. hellem* (30:1/spores: cells) for 24 h. The controls were either uninfected or infected by *S. aureus* (5:1/DCs: *S. aureus*). These DCs were washed with PBS to get rid of excess pathogens, and then, Jurkat cells were added to the culture groups (1:1/DCs: T cells). The innate immune cells and the lymphocytes were co-incubated for 12 h. After that, the flasks were gently stirred to collect the suspension cells (T cells); the bottom-attached cells were collected as DCs and were used for further analysis.

### Flow cytometry analysis

The immune cell profiles, cell characterization, and the expressions of cell surface markers were assessed by flow cytometry analysis. Single-cell suspensions, from various treatments, were washed with 1x PBS/0.3% BSA and then stained with fluorochrome-conjugated antibodies (all purchased from BioLegend) for 30 min at 4°C. Samples were subjected to analysis via FACSCanto II flow cytometer (BD Biosciences), and the data were analyzed with FACSDiva software (v6.1.2).

### Cytokine expressions

The expression levels of cytokines such as IL-6 and IL-12 were detected by ELISA kits (Thermo Fisher Scientific). Samples were either mouse plasma or cell culture supernatants. The mouse peripheral blood was drawn with the addition of anticoagulant sodium citrate, and the blood samples were then centrifuged at 400*g* for 10 min to get the supernatant plasma.

### qRT-PCR

To assess transcriptional levels of target genes, total RNAs of different cells such as DCs would be extracted by TRIzol (Ambion). The RNA samples were then reverse-transcribed to cDNAs using High-Capacity RNA-to-cDNA Kit (Yeasen Biotechnology). The qRT-PCR assay was carried out according to the Hieff qRT-PCR SYBR Green Master Mix instructions (Yeasen Biotechnology). The gene/primer information is shown in supplementary data (Table S3).

Table S3 Primers for qRT-PCR analysis.

### RNA interference assay

siRNA specifically designed for targeting DCs’ *NFAT5* was synthesized by Sangon Biotech Co., Ltd, using the primers set, sense 5′-CCAGUUCCUACAAUGAUAACACUGA-3′ and anti-sense 5′-UCAGUGUUAUCAUUGUAGGAACUGG-3′. The siRNAs were then isolated and purified. To block the expression of *NFAT5*, 4 μl of the interference fragment was mixed with 8 μl of INTERFERin siRNA transfection reagent in 200 μl of low-serum Opti-MEM, incubated at room temperature for 10 min, and then evenly added dropwise to cells containing 2 ml of complete RPMI 1640 (Gibco). Cells were cultured in a 37°C, 5% CO_2_ incubator for 6 h until the medium was refreshed, and cells were kept culturing in complete RPMI 1640 supplemented with 10% FBS in a 37°C, 5% CO_2_ incubator.

### Immunofluorescent assays

Subcellular localization and translocalization of NFAT5 were assessed by anti-NFAT5 antibody (Cat: ab3446; Abcam). DCs were either infected by *E. hellem* (30:1/spores: cells) or *S. aureus* (5:1/DCs: *S. aureus*), or stimulated by NaCl (110 mM). Cells were fixed by paraformaldehyde and permeabilized with Triton X-100. After washing and blocking, samples were incubated with NFAT5 antibody, followed by Alexa Fluor 594–conjugated secondary antibody (Thermo Fisher Scientific). Samples were then washed in PBST and stained for 5 min in DAPI (4′,6-diamidino-2-phenylindole; Sigma-Aldrich) for nucleus labeling. Samples were imaged using an Olympus FV1200 laser scanning confocal microscope (Olympus).

### Label-free quantitative mass spectrometry

The total proteins of DC2.4 cells, either uninfected or infected by *E. hellem*, were extracted. The protein samples were then subjected to label-free quantitative mass spectrometry analysis performed on a Q Exactive mass spectrometer (Thermo Fisher Scientific). The MS data were analyzed using MaxQuant software, version 1.5.3.17 (Max Planck Institute of Biochemistry in Martinsried, Germany). The biological functions of proteins were annotated by Gene Ontology (GO) Annotation (Blast2GO; http://www.blast2go.com) and the online KEGG database (http://geneontology.org/). Differentially expressed proteins between the two groups were screened using fold change greater than 1.5-fold (up to 1.5-fold or less than 0.5). Those selected proteins were explored after bioinformatic analysis, including hierarchical cluster by ComplexHeatmap R (version 3.4), KEGG functional enrichment analysis, and protein–protein interaction network using the IntAct molecular interaction database (http://www.ebi.ac.uk/intact/). Statistical significance was analyzed using a *t* test based on *P*-value < 0.05.

### Statistics

Statistical analysis of results was conducted by a *t* test or two-way ANOVA and to identify the differences between two groups, with *P* < 0.05 being considered a significant difference.

## Data Availability

The datasheet about mass spectrometry identification of top differentially expressed proteins is included in the supplementary materials of the study. All other details and data were available upon request.

## Supplementary Material

Reviewer comments
